# Anti-Inflammatory Effects of *Spirulina platensis* Extract via the Modulation of Histone Deacetylases

**DOI:** 10.3390/nu8060381

**Published:** 2016-06-21

**Authors:** Tho X. Pham, Young-Ki Park, Ji-Young Lee

**Affiliations:** Department of Nutritional Sciences, University of Connecticut, Storrs, CT 06269, USA; tho.pham@uconn.edu (T.X.P.); young-ki.park@uconn.edu (Y.-K.P.)

**Keywords:** *Spirulina platensis*, histone deacetylases, anti-inflammatory, inflammation, epigenetics

## Abstract

We previously demonstrated that the organic extract of *Spirulina platensis* (SPE), an edible blue-green alga, possesses potent anti-inflammatory effects. In this study, we investigated if the regulation of histone deacetylases (HDACs) play a role in the anti-inflammatory effect of SPE in macrophages. Treatment of macrophages with SPE rapidly and dose-dependently reduced HDAC2, 3, and 4 proteins which preceded decreases in their mRNA levels. Degradation of HDAC4 protein was attenuated in the presence of inhibitors of calpain proteases, lysosomal acidification, and Ca^2+^/calmodulin-dependent protein kinase II, respectively, but not a proteasome inhibitor. Acetylated histone H3 was increased in SPE-treated macrophages to a similar level as macrophages treated with a pan-HDAC inhibitor, with concomitant inhibition of inflammatory gene expression upon LPS stimulation. Knockdown of HDAC3 increased basal and LPS-induced pro-inflammatory gene expression, while HDAC4 knockdown increased basal expression of interleukin-1β (IL-1β), but attenuated LPS-induced inflammatory gene expression. Chromatin immunoprecipitation showed that SPE decreased p65 binding and H3K9/K14 acetylation at the *Il-1β* and tumor necrosis factor α (*Tnfα*) promoters. Our results suggest that SPE increased global histone H3 acetylation by facilitating HDAC protein degradation, but decreases histone H3K9/K14 acetylation and p65 binding at the promoters of *Il-1β* and *Tnfα* to exert its anti-inflammatory effect.

## 1. Introduction

Chronic inflammation is causally linked to the pathogenesis of obesity-induced metabolic diseases, such as insulin resistance, type 2 diabetes, cardiovascular disease (CVD) and non-alcoholic fatty liver disease [1,2]. Pro-inflammatory cytokines, including tumor necrosis factor α (TNFα), interleukin-6 (IL-6) and interleukin-1β (IL-1β), have shown to deteriorate normal cellular functions, leading to the metabolic diseases [3,4].

Recent studies utilizing inhibitors and genetic ablation of histone deacetylases (HDACs) have suggested that HDACs are obligatory for optimal induction of inflammatory gene expression in macrophages [5,6,7,8]. Therefore, HDAC inhibitors have emerged as anti-inflammatory agents. HDACs are epigenetic regulators of gene transcription by catalyzing the removal of acetyl moieties from lysine residues on histone tails, which is generally associated with transcriptional repression [9]. Eighteen HDACs have been identified in mammals to date and they are categorized into two families: the classical zinc-dependent HDACs of the Rpd3/Hda1 family and the NAD^+^-dependent sirtuin family [10]. HDACs are also classified based on their phylogeny. Class I HDACs, *i.e.*, HDAC1, 2, 3 and 8, are homologous to yeast (*Saccharomyces cerevisiae*) RPD3 protein. Class II HDACs are similar to yeast HDA1 and are further subcategorized into class IIa (HDAC4, 5, 7, and 9) and IIb (HDAC6 and 10). Class III HDACs are sirtuin 1–7 and class IV is HDAC11 [11,12]. Abnormalities in HDACs have been implicated in several human diseases including, but not limited to, obesity, diabetes, and CVD [13,14,15]. Furthermore, HDACs are suggested to play a vital role in both innate and adaptive immunity [16].

*Spirulina platensis* (SP) is a multicellular and filamentous edible blue-green alga that is found naturally in alkaline lakes. It has also been cultured in a controlled environment for human consumption. The protective effects of SP against inflammatory diseases, such as colitis, arthritis, and allergic rhinitis, have been documented in animals and humans, although the molecular mechanisms are not well understood [17,18,19]. We previously reported that IL-6 secretion from the splenocytes isolated from apolipoprotein E knockout mice fed a diet supplemented with SP was decreased upon lipopolysaccharide (LPS) challenge [20]. Furthermore, we also found that the organic extract of SP (SPE) represses TNFα expression and secretion of pro-inflammatory cytokines with concomitant increases in histone H3 acetylation in macrophages [20]. In the present study, we sought to determine if the anti-inflammatory effect of SPE is attributable to a change in histone H3 acetylation via the modulation of HDAC expression in macrophages.

## 2. Materials and Methods

### 2.1. SPE Preparation

SP powder (Earthrise^®^ Natural Spirulina) was kindly provided by Earthrise Nutritionals (Irvine, CA, USA) and extracted into chloroform/methanol (1:2) as we previously described [21,22]. The extract was stored under N_2_ gas at −20 °C for short term and −80 °C for long term. To incorporate SPE into cell culture medium, SPE was dried down under N_2_ to completely remove any solvents and then dissolved in dimethyl sulfoxide (DMSO) (0.5% DMSO final concentration). For all experiments, 0.5% DMSO vehicle control was run in parallel.

### 2.2. Bone Marrow Isolation and Macrophage Differentiation

Mouse bone marrow-derived macrophages (BMDM) were differentiated from bone marrow isolated from the tibia and femur of C57BL/6J mice (Jackson Laboratory, Bar harbor, ME, USA). The bone marrow from each limb was pooled and differentiated into macrophages for experiments as we previously described [20,23]. All animal procedures were approved by the Institutional Animal Care and Use Committee of the University of Connecticut (A13-026).

### 2.3. Cell Culture and Treatments

RAW 264.7 macrophages (RAW macrophages) were purchased from ATCC (Manassas, VA, USA). RAW macrophages were maintained in RPMI-1640 containing 10% fetal bovine serum (FBS), 1x vitamin mix, 100 U/mL penicillin, 100 μg/mL streptomycin, and 2 mmol/L l-glutamine. Cells were kept in a humidified incubator at 37 °C with 5% CO_2_. Cells were treated with SPE (0–100 μg/mL) for an indicated time, after which they were activated with LPS (100 ng/mL). For experiments using tricostatin A (TSA), a pan-HDAC inhibitor, RAW macrophages were pretreated with TSA at a concentration of 25 or 100 nmol/L for 12 h, followed by another 18 h of TSA in the presence or absence of LPS. All cell culture supplies were purchased from Hyclone (Logan, UT, USA).

### 2.4. HDAC3 and 4 Knockdown by Small Interfering RNA (siRNA)

RAW macrophages were transfected with Silencer^®^ Negative Control scrambled siRNA (Ambion, Invitrogen, Grand Island, NY, USA) or siGENOME™ SMARTpool HDAC3 siRNA or HDAC4 siRNA (GE Healthcare Dharmacon, Lafayette, CO, USA) as we described previously [24]. Twenty four hours after the transfection, cells were stimulated with 100 ng/mL LPS for 3 h for subsequent gene analysis.

### 2.5. Quantitative Real-Time PCR (qRT-PCR)

Total RNA was extracted using TRIzol^®^ RNA Isolation Reagent (Life Technologies, Carlsbad, CA, USA) following the manufacturer’s protocol. Reverse transcription for cDNA synthesis and qRT-PCR analysis was performed as previously described [20,25]. Primers were designed using Beacon Designer (Premier Biosoft, Palo Alto, CA, USA) and the sequences will be available upon request.

### 2.6. Chromatin Immunoprecipitation (ChIP)

RAW macrophages were pretreated with vehicle control (DMSO) or 100 μg/mL SPE for 12 h, and then stimulated with 100 ng/mL LPS for 18 h. Subsequently, the cells were washed and fixed with 1% formaldehyde in PBS. The cells were then harvested and lysed with an L1 buffer supplemented with protease inhibitors. The cell lysates were sonicated on wet ice using a Misonix sonicator (Farmingdale, NY, USA) for six of 15-s pulses with 1 min intervals at a power setting of 4. Subsequently, the chromatin was aliquoted and kept at −80 °C until use. For each immunoprecipitation (IP), 10 μg of chromatin was diluted in low ionic strength ChIP dilution buffer and 10 μL of the chromatin preparation was kept as an input control. All antibodies used for IP, including nuclear factor κB (NF-κB) p65, acetylated histone H3K9/K14 and IgG, were purchased from Cell Signaling Technology (Danvers, MA, USA). Chromatin was immunoprecipitated with one of the aforementioned antibodies on a rotator overnight at 4 °C. The next day, the chromatin and antibody complex was incubated with 60 μL of protein A dynabead (Life Technologies, Carlsbad, CA, USA) that was prebinded with salmon testes for 4 h at 4 °C on a rotator. The complex of dynabead, antibody, and chromatin was then pulled down using a 6-tube magnetic stand (Applied Biosystems, Foster City, CA, USA) and washed sequentially with high salt buffer (500 mM NaCl, 0.1% SDS, 1% Triton X-100, 2 mM EDTA pH 8.0, 20 mM Tris-Cl pH 8.1), lithium chloride buffer (0.25 M LiCl, 1% IGEPAL, 1% Deoxycholic acid, 1 mM EDTA pH 8.0, 10 mM Tris-HCl, pH 8.1), and Tris-EDTA buffer (10 mM Tris-Cl pH 8.0, 1 mM EDTA) for 5 min each at 4 °C. Subsequently, the antibody and chromatin complex was eluted from dynabeads using elution buffer (90 mM NaHCO_3_, 1% SDS) for 15 min at room temperature. The eluate containing antibody and chromatin was then reverse cross-linked with a decrosslinking buffer (200 mM NaCL, 10 mM ETDA pH 8.0, 50 mM Tris-HCl pH 6.8) containing 2.5 μL of proteinase K (20 mg/mL) (Life Technologies, Carlsbad, CA, USA) at 65 °C for 4 h. The DNA was then purified using a QIAquick^®^ PCR purification kit (Qiagen, Velencia, CA, USA). The purified DNA was then quantified using a CFX96 real time machine (Bio-Rad, Hercules, CA, USA). Primer sequence will be available upon request.

### 2.7. Western Blot Analysis

Histones and whole cell lysates were prepared as we previously described [20] and Western blot analysis was conducted as described [21,22]. Protein concentrations were quantified using a bicinchoninic acid assay kit (Thermo Fisher Scientific, Grand Island, NY, USA). The following antibodies were used: HDAC2, 3, and 4 (Santa Cruz Biotechnology, Santa Cruz, CA, USA); HDAC4 (Cell Signaling, Danvers, MA, USA); histone H3 and acetylated H3 (Abcam, Cambridge, MA, USA); and β-actin (Sigma-Aldrich, St. Louis, MO, USA) for a loading control. Blots were developed using a Westpico horseradish peroxidase chemiluminescence (Thermo Fisher Scientific, Grand Island, NY, USA) and imaged using a Chemidoc XRS+ system (Bio-Rad, Hercules, CA, USA) and Image Lab software (Bio-Rad).

### 2.8. Statistical Analysis

One-way analysis of variance (ANOVA) and Newman-Keuls *post hoc* analysis or unpaired *t*-test were performed with Graphpad Prism 6.0 (GraphPad Software, La Jolla, CA, USA) to detect differences between group means. *P* values less than 0.05 were considered significant. Values were expressed as mean ± SEM.

## 3. Results

### 3.1. Reduction in HDAC2, 3 and 4 *Proteins* by SPE Preceded Decreases in Their mRNA Levels in Macrophages

**Figure 1:**
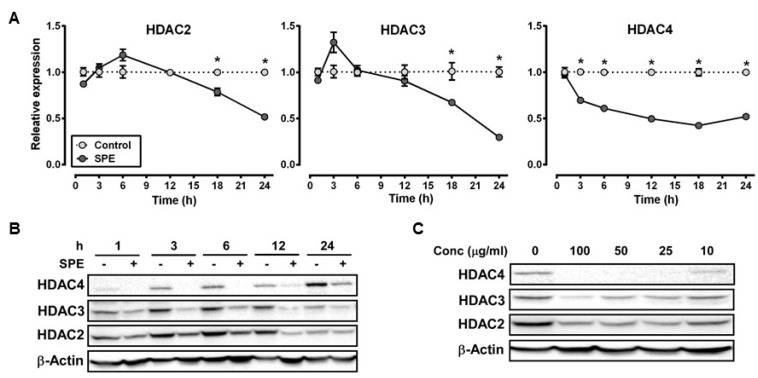
*Spirulina platensis* (SPE) reduce histone deacetylase (HDAC) mRNA expression and protein in a time and dose dependent manner in RAW 264.7 macrophages. (**A**) mRNA expression of HDACs in RAW 264.7 macrophages treated with 100 μg/mL of SPE for indicated amount of hours. *N* = 3, value = mean ± SEM * indicates significantly different from control (*p* < 0.05); (**B**) Western blot time course and (**C**) dose-response of HDACs in RAW 264.7 macrophages treated with 100 μg/mL of SPE for indicated amount of time or varying SPE concentrations. A represented blot of three independent experiments is shown.

**Figure 2:**
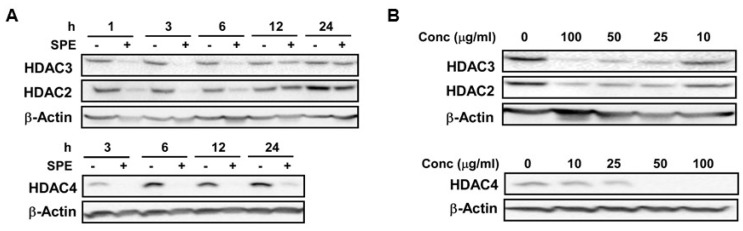
SPE reduced HDAC proteins in a dose and time-dependent manner in bone marrow-derived macrophages (BMDM). (**A**) Western blot analysis of HDAC2, 3, and 4 in BMDM treated with 100 μg/mL of SPE for indicated amount of time; (**B**) Western blot analysis of HDAC2, 3, and 4 in BMDM treated with indicated concentration of SPE for 3 h. A representative blot of 2–3 experiments is shown.

We selected HDAC2 and 3, and HDAC4 as representative of class I and class II HDACs, respectively, for dose-dependent and time-course experiments to measure the effect of SPE on their mRNA and protein abundance in RAW macrophages. The mRNA abundance of HDAC2 and 3 was not significantly altered by SPE until 18 h of incubation, at which HDAC2 and 3 mRNA levels were significantly decreased (Figure 1A). Compared with HDAC2 and 3, HDAC4 mRNA was rapidly decreased by SPE with a significant difference from 3 h of SPE incubation. At the protein level, SPE reduced the abundance of HDAC4 and to a lesser degree HDAC2 and 3 as early as 1 h of treatment in RAW macrophages (Figure 1B). Treatment of SPE at concentrations of 10, 25, 50, or 100 μg/mL for 3 h reduced protein levels of HDAC2 and 3 in macrophages in a dose-dependent manner (Figure 1C). In contrast to these HDACs, concentrations of 25 μg/mL or higher of SPE lowered HDAC4 protein to an undetectable level in RAW macrophages. To confirm that the effects of SPE was not cell-line specific, dose-dependent and time-course experiments for HDAC2, 3 and 4 protein abundance were performed in BMDM. Similar to RAW macrophages, SPE in a time- and dose-dependent manner reduced the HDAC protein levels in BMDM (Figure 2A,B).

### 3.2. The Lysosome, Calpain Proteases, and Ca^2+^/Calmodulin-Dependent Protein Kinase II Are Involved in the Rapid HDAC4 Degradation by SPE

**Figure 3:**
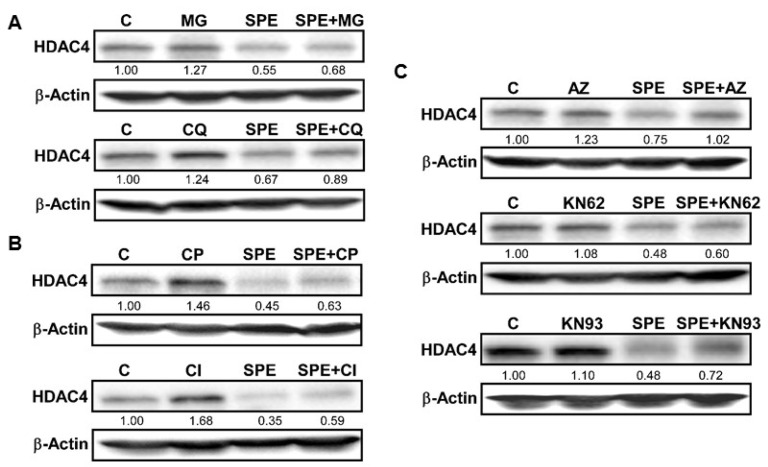
Lysosomal and calpain-mediated degradation of HDAC4 by SPE. (**A**) Western blot analysis of HDAC4 in RAW 264.7 macrophages pretreated with 10 μg/mL of MG-132 (top), or 50 μM of chloroquine (bottom) for 1 h and then treated with 25 μg/mL of SPE in the presence of inhibitors; (**B**) Western blot analysis of HDAC4 in RAW 264.7 macrophages pretreated with calpeptin (10 μg/mL) for 1 h and then treated with 25 μg/mL of SPE in the presence of inhibitors; (**C**) Western blot analysis of HDAC4 in RAW 264.7 macrophages pretreated with CAMKII inhibitor KN-93 at the concentration of 5 μM for 1 h and then treated with 25 μg/mL of SPE in the presence of inhibitors. A represented blot of three independent experiments is shown.

HDAC4 protein was rapidly reduced by SPE, much faster than HDAC2 and 3. Therefore, we investigated potential mechanisms of action for HDAC4 protein degradation. Two major protein degradation pathways in mammalian cells involve proteasomes and lysosomes. We used MG-132 (MG) and chloroquine (CQ), inhibitors of the proteasome and lysosomal acidification, respectively, to evaluate the role of proteasomes and lysosomes in HDAC4 degradation facilitated by SPE in RAW macrophages. MG was not able to counteract the decrease in HDAC4 protein by SPE and in fact, HDAC4 protein was almost non-detectable in the presence of MG (Figure 3A). When macrophages were incubated with CQ, the inhibitor modestly increased basal HDAC4 protein levels and attenuated SPE-induced HDAC4 protein reduction although it did not completely abolish the SPE effect. HDAC4 has been reported to contain a PEST-domain, which is recognized by calpain proteases [26]. Calpeptin (CP), an inhibitor of calpain proteases, noticeably increased basal HDAC4 protein in RAW macrophages and also attenuated SPE-induced HDAC4 reduction (Figure 3B). As Ca^2+^ is an activator of calpain proteases, we also explored the role of Ca^2+^/calmodulin-dependent protein kinase II (CAMKII), which has been reported to phosphorylate HDAC4 [27]. Treatment of RAW macrophages with KN93, a CAMKII inhibitor, was able to attenuate the reduction of HDAC4 by SPE to a certain degree (Figure 3C).

### 3.3. SPE Increased Acetylated Histone H3 in RAW Macrophages

**Figure 4:**
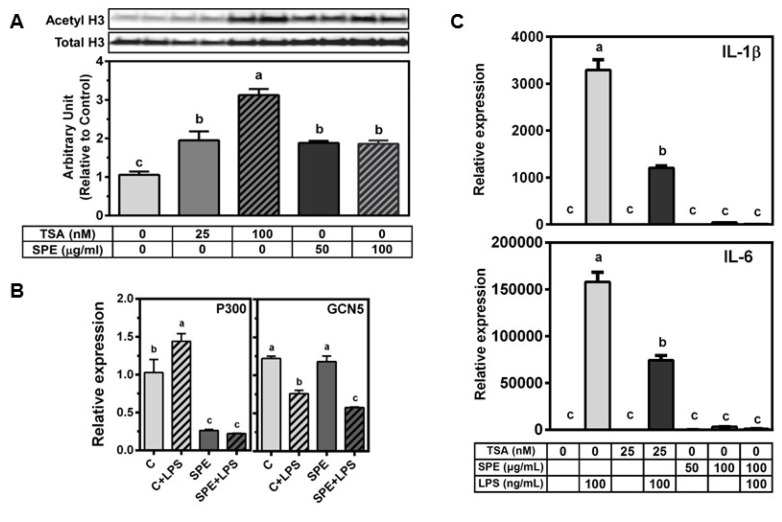
SPE increase acetylated histone H3 similarly to tricostatin A (TSA). (**A**) Western blot analysis of acetylated histone H3K9 in RAW 264.7 macrophages. Cells were pretreated with vehicle control (0.5% DMSO), 50 or 100 μg/mL of SPE for 12 h. The cells were then treated with vehicle control (0.5% DMSO), 25 nM TSA, 100 nM TSA, 50 or 100 μg/mL of SPE for 18 h; Blot image (top) and quantification (bottom) (**B**) mRNA expression of histone acetyltransferase p300 and GCN5 in RAW 264.7 macrophages pretreated with vehicle control (0.5% DMSO), or 100 μg/mL of SPE for 12 h and then stimulated with 100 ng/mL LPS for 18 h; *n* = 3 (**C**) mRNA expression of inflammatory genes in RAW 264.7 macrophages pretreated with vehicle control (0.5% DMSO), 25 nM TSA, 50, or 100 μg/mL of SPE for 12 h. Cells were then treated with vehicle control, 25 nM TSA, 50 or 100 μg/mL SPE alone or in combination with 100 ng/mL of LPS for 18 h. Different letters indicate significantly different (*p* < 0.05). Mean ± SEM, *n* = 3. Bars with different letters are significantly different (*p* < 0.05).

TSA, a pan-HDAC inhibitor, at 25 or 100 nmol/L dose-dependently increased acetylated histone H3 in RAW macrophages (Figure 4A). When RAW macrophages were incubated with 50 or 100 μg/mL of SPE, there was a significant increase in acetylated histone H3 to a similar level of 25 nmol/L TSA treatment. As the levels of acetylated histones are determined by opposing actions of HDACs and histone acetyltransferases (HATs), we also measured if SPE alters the expression of two common HATs, *i.e.*, p300 and general control non-derepressible 5 (GCN5), in RAW macrophages. LPS significantly increased p300 mRNA but SPE markedly decreased p300 mRNA abundance both in unstimulated and LPS-stimulated macrophages (Figure 4B). In contrast, LPS significantly decreased GCN mRNA in control and SPE-treated cells.

As we observed that TSA at 25 nmol/L and SPE at 50 and 100 μg/mL increased the level of acetylated histone H3 to the similar degree in RAW macrophages, we compared the effect of TSA and SPE on LPS-induced pro-inflammatory gene expression. TSA significantly inhibited LPS-induced IL-1β and IL-6 by ~50%–65% (Figure 4C) while SPE at 100 μg/mL almost completely abrogated the induction of the pro-inflammatory genes by LPS to the level of unstimulated macrophages.

### 3.4. HDAC3 Deficiency Increased, While HDAC4 Deficiency Attenuated LPS-Induced Inflammatory Gene Expression in RAW Macrophages

**Figure 5:**
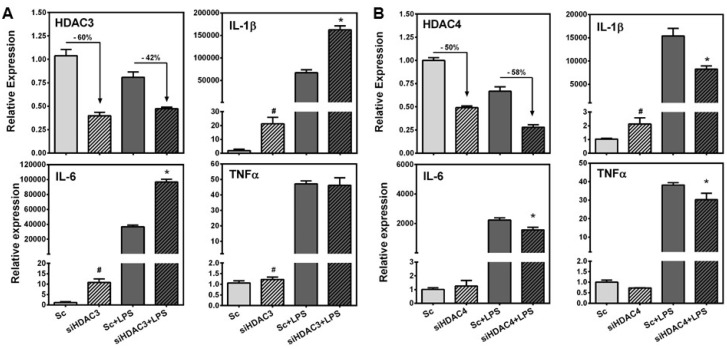
Effects of HDAC3 and 4 knockdown on LPS-induced inflammatory gene expression. RAW 264.7 macrophages were transfected with scrambled control, siRNA against HDAC3 or HDAC4 for 24 h and then cells were stimulated with or without 100 ng/mL of LPS for 3 h for subsequent gene expression qRT-PCR. (**A**) Percent knockdown of HDAC3 (top left), expression of IL-1β (top right), expression of IL-6 (bottom left), and expression of TNFα (bottom right); (**B**) Percent knockdown of HDAC4 (top left), expression of IL-1β (top right), expression of IL-6 (bottom left), and expression of TNFα (bottom right). ). ^#^ indicates significantly different from scrambled control (*p* < 0.05); * indicates significantly different from scrambled control + LPS (*p* < 0.05).

As SPE repressed the expression of HDAC3 and 4 at the transcriptional as well as post-transcriptional levels, we investigated potential effects of the HDAC repression on pro-inflammatory gene expression using siRNA. Knockdown of HDAC3 and 4 in RAW macrophages by siRNA reduced their expression by ~50%–60% (Figure 5). Compared to scrambled control, HDAC3 knockdown significantly increased basal expression of IL-1β, IL-6, and TNFα in unstimulated macrophages while HDAC4 knockdown only increased the basal expression of IL-1β. In LPS-stimulated RAW macrophages, HDAC3 deficiency significantly increased the expression of IL-1β and IL-6, but not TNFα, while a reduction in HDAC4 attenuated LPS-induced IL-1β, IL-6 and TNFα expression compared to LPS-stimulated scrambled control.

### 3.5. SPE Reduced p65 Binding and H3 Acetylation in the Il-1β and Tnfα Promoter

**Figure 6:**
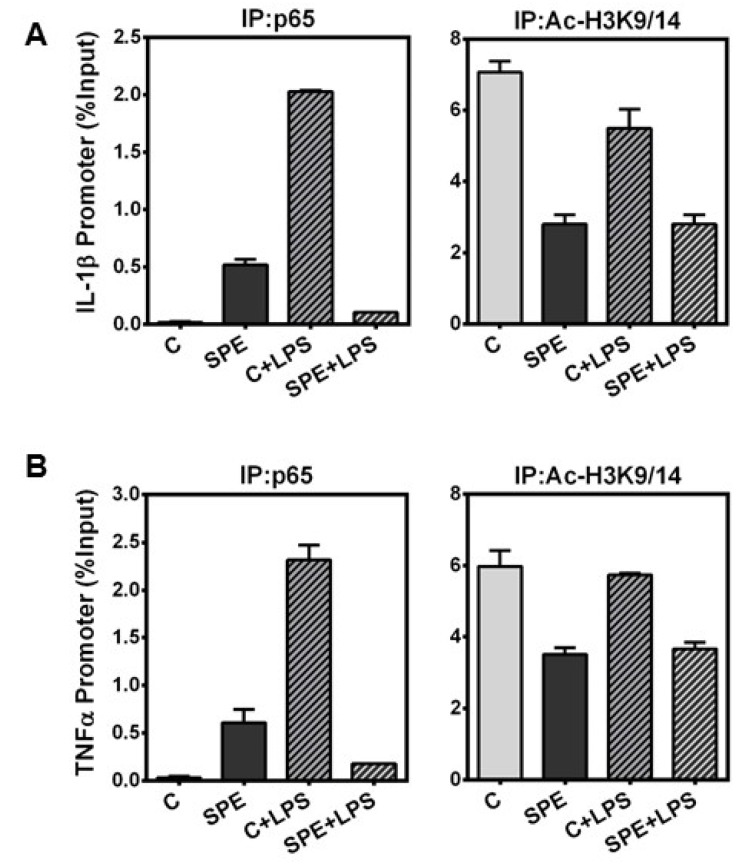
Chromatin Immunoprecipitation of p65 and H3K9/K14 at the promoter of inflammatory genes. ChIP of RAW 264.7 macrophages pretreated with 100 μg/mL of SPE for 12 h and then stimulated with 100 ng/mL of LPS for an additional 18 h in the presence of SPE. ChIP DNA was obtained by immunoprecipitation of p65 (left) and histone H3K9 (right) and quantified by qPCR with primers at the (**A**) IL-1β promoter and (**B**) TNFα promoter.

Histone hyperacetylation is associated with transcriptionally-active genes and an open chromatin state allowing for transcription factor binding, while hypoacetylation is associated with transcriptionally inactive, condensed chromatin [28,29]. We observed that SPE treatment in macrophages increased histone H3 acetylation, but paradoxically repressed inflammatory gene expression. We therefore performed ChIP analysis to determine whether NF-κB p65, a major transcription factor responsible for IL-1β and TNFα transcription [30], was able to bind the *Il-1β* and *Tnfα* promoter. Furthermore, we determine the acetylation state of chromatin at the *Il-1β* and *Tnfα* promoter. Stimulation of RAW macrophages with LPS increased p65 binding to *Il-1β* and *Tnfα* promoter, which was abrogated by SPE (Figure 6A). ChIP with anti-acetylated H3K9/14 showed that SPE reduced the acetylation of histone H3 at the promoters of *Il-1β* and *Tnfα* regardless of LPS stimulation (Figure 6B).

## 4. Discussion

Dysregulated inflammation contributes to the development of inflammatory, metabolic diseases such as atherosclerosis, non-alcoholic fatty liver disease, and diabetes [31]. Therefore, it is important to identify anti-inflammatory agents for the prevention/therapy for the inflammatory diseases. We previously demonstrated that SPE decreases the expression and secretion of pro-inflammatory cytokines in LPS-stimulated macrophages via the inhibition of nuclear translocation of NF-κB p65 [20]. However, this study also suggested the presence of additional anti-inflammatory mechanisms. As modulators of the epigenome, HDACs control the transcription of a myriad set of genes [11]. In particular, studies have shown that HDACs play a critical role in the regulation of inflammation [5,8]. However, the effect of individual HDAC isoforms on the regulation of inflammatory pathways remains largely unknown. Furthermore, whether bioactive compounds from food or natural products can alter the expression/functions of HDACs to exert their biological effects has not been well studied. In the present study, we found that the anti-inflammatory effects of SPE are associated with changes in mRNA and/or protein levels of HDACs and resultant alterations in histone acetylation state in macrophages.

We found that SPE dramatically decreased HDAC2, 3 and 4 protein levels in a time and dose-dependent manner in two macrophage models, *i.e.*, RAW macrophages and mouse BMDM. The reduction of class I HDACs, *i.e.*, HDAC2 and 3, was much slower than HDAC4, a class II HDAC. Also, the decrease of HDAC proteins by SPE preceded that of their mRNA levels, suggesting that transcriptional regulation is unlikely to be responsible for the rapid decrease in the HDAC proteins at early time points. The major pathways for protein degradation in cells involve proteasomes and autophagy/lysosomes [32]. Our data indicate that the lysosomal degradation appears to play a role in SPE-induced HDAC4 degradation while proteasomes minimally contribute to the degradation. We found that calpain protease and CAMKII may also be involved in SPE-induced HDAC4 degradation. As CAMKII has been demonstrated to phosphorylate HDAC4, leading to its cytoplasmic localization [27], it is presumable that SPE may increase CAMKII activity, which leads to HDAC4 cytoplasmic localization and subsequent cleavage by calpain proteases and lysosomal degradation. Future study is necessary to gain more insight into how SPE modulates CAMKII activity in macrophages and if the action is required for exerting its anti-inflammatory effects.

The main substrates for HDACs are lysine residues on histone tails although HDACs have been shown to deacetylate other protein targets [9]. Due to the rapid protein degradation and transcriptional repression of HDACs by SPE, acetylated histone H3 was significantly increased by SPE to a similar extent to that of 25 nmol/L of TSA in macrophages. SPE significantly decreased the expression of p300, but did not alter GCN5 expression. Therefore, although we cannot rule out that other HATs may be involved, it is presumable that HDAC degradation rather than increased HAT activity is responsible for elevated levels of acetylated histone H3 by SPE.

We demonstrated that both SPE and TSA increased histone H3 acetylation to similar levels, although SPE was a more potent inhibitor of LPS-stimulated inflammatory gene expression. One possible explanation is that compensatory up-regulation of HDAC mRNA expression upon TSA treatment (data not shown) may restore HDAC protein to a certain degree, whereas SPE can decrease HDAC transcription as well as facilitate protein degradation. As both TSA and SPE increased the levels of acetylated histone H3, a mark for open chromatin and active transcription, it may be counterintuitive to find the concomitant repression of pro-inflammatory genes. Our ChIP analysis showed that despite an increase in global acetylated histone H3 by SPE, SPE in fact, reduced histone H3K9/K14 acetylation in the promoters of *Il-1β* and *Tnfα*. The decrease in H3 acetylation may be responsible for diminished p65 binding to the promoters of *Il-1β* and *Tnfα* as shown in ChIP analysis. It is also possible that the inhibitory action of SPE on HDACs may affect inflammation, in part, through a histone-independent mechanism considering that acetylated histone H3 levels were similar between 25 nmol/L TSA and SPE despite more potent anti-inflammatory effects of SPE than TSA.

Both HDAC3 and HDAC4 have been shown to associate with NF-κB p65 [33,34,35]. It was proposed that deacetylation of p65 by HDAC3 promotes its binding to the inhibitor of κB, resulting in the export of NF-κB from the nucleus and down-regulation of NF-κB target gene expression [34]. HDAC4 has also been suggested to limit NF-κB through a similar mechanism [33]. The present study supports that HDAC3 limits NF-κB activation, because HDAC3 knockdown increased basal and LPS-stimulated expression of inflammatory genes in macrophages. We also found that knockdown of HDAC4 increased the basal expression of IL-1β, suggesting that it participate in the active repression of IL-1β, but not IL-6 and TNFα. Surprisingly, we observed that HDAC4 knockdown attenuated LPS-induced inflammatory gene expression. This suggests that although HDAC4 limits NF-κB activity in the unstimulated state, it may play a role in TLR4 signaling that may be disturbed in macrophages with HDAC4 deficiency. The suggestion that both HDAC3 and HDAC4 limit NF-κB activity appears to counter our speculation that SPE mediates its anti-inflammatory effect through the degradation of HDAC3 and 4. However, studies in the literature suggest that reduction of HDAC3 may not entirely increase inflammation. *Hdac3* knockout macrophages displayed a phenotype similar to alternatively activated M2 macrophages, which possess anti-inflammatory properties [7]. Furthermore, *Hdac3* knockout macrophages were unable to activate almost half of their inflammatory gene expression program in response to LPS stimulation [8]. While our study using HDAC3 siRNA and others [34] have demonstrated that HDAC3 possess an anti-inflammatory properties, findings from *Hdac3* knockout macrophages support that HDAC3 possesses pro-inflammatory effects. The discrepancy between the two interpretations may be due to differences between knockdown and knockout experiments. Further studies are warranted to have a better understanding of the role of HDACs in inflammation.

## 5. Conclusions

Taken together, our present study demonstrated that SPE facilitates the protein degradation of several HDAC isoforms and inhibits their mRNA transcription, consequently increasing global acetylated histone H3 in macrophages. It is of interest, however, that SPE inhibited histone H3 acetylation on the promoters of pro-inflammatory genes, suggesting that non-histone targets of SPE-regulated HDACs likely exist in macrophages. Regardless, the repression of HDACs may contribute, at least in part, to the potent anti-inflammatory effect of SPE. Our study underscores a new avenue of mechanisms of action by which natural products exert epigenetic regulation for the anti-inflammatory effects in macrophages.

## Abbreviations

The following abbreviations are used in this manuscript:

BMDM	bone marrow-derived macrophage
CVD	cardiovascular disease
GCN5	general control non-derepressible 5
HAT	histone acetyltransferase
HDAC	histone deacetylase
IL-1β	interleukin-1β
IL-6	interleukin-6
LPS	lipopolysaccharide
NF-κB	nuclear factor-κB
SP	*Spirulina platensis*
SPE	*Spirulina platensis* organic extract
TLR4	toll-like receptor 4
TNFα	tumor necrosis factor α
TSA	trichostatin A
